# N170 Changes Show Identifiable Chinese Characters Compete Primarily with Faces Rather than Houses

**DOI:** 10.3389/fpsyg.2015.01952

**Published:** 2016-01-05

**Authors:** Cong Fan, Weiqi He, Huamin He, Guofang Ren, Yuejia Luo, Hong Li, Wenbo Luo

**Affiliations:** ^1^Research Center of Brain and Cognitive Neuroscience, Liaoning Normal UniversityDalian, China; ^2^Laboratory of Cognition and Mental Health, Chongqing University of Arts and SciencesChongqing, China; ^3^School of Education Science, Anyang Normal UniversityAnyang, China; ^4^Institute of Affective and Social Neuroscience, Shenzhen UniversityShenzhen, China

**Keywords:** N170, identifiable Chinese characters, faces, houses, competition

## Abstract

Character processing is a crucial cognitive skill that is highly emphasized and industriously cultivated in contemporary society. In the present study, using a competition paradigm, we examined the electrophysiological correlates of different relationships between Chinese characters and faces and between Chinese characters and houses during early visual processing. We observed that identifiable Chinese characters compete primarily with faces rather than houses at an early visual processing stage, with a significantly reduced N170 for faces but not for houses, when they were viewed concurrently with identifiable characters relative to when they were viewed concurrently with unidentifiable characters. Consistent with our previous study, there was a significant increase in N170 after characters have been learned, indicating a modulatory effect of Chinese character identification level on N170 amplitude. Furthermore, we found an enlarged N170 in response to faces compared to houses, indicating that the neural mechanisms for processing faces and houses are different at an early visual processing stage.

## Introduction

The invention of characters is one of the important events marking the beginning of human civilization. Character processing is a crucial cognitive skill that is highly emphasized and industriously cultivated in modern society. Since writing emerged just 5,400 years ago (Dehaene and Cohen, 2007), character processing was not an original function of the human visual system. Furthermore, the neural resources of the human brain are limited (Slagter et al., 2007; Schroer, 2008). These two points raise questions about whether the human brain can reallocate its resources for character processing.

The neuronal recycling hypothesis (Dehaene and Cohen, 2007) may explain such resource reallocation mechanisms. It emphasizes that writing emerged so recently in evolutionary terms that there could not have been any genetic changes. Therefore, written language processes invade and reuse cortical areas with pre-existing functions during education (Dehaene, 2005, 2009; Dehaene and Cohen, 2007). Based on this viewpoint, recent studies have provided neuroimaging and electrophysiological evidences showing that as the processing facility of written languages increases, written language processing recruits face-related processes, resulting in a competition between the two events (Dehaene et al., 2010; Li et al., 2013; Fan et al., 2015).

Functional magnetic resonance imaging (fMRI) studies have demonstrated decreased responses in visual word form area (VWFA), which is responsible for processing visual words (McCandliss et al., 2003; Bolger et al., 2005; Dien, 2009; Thesen et al., 2012), to faces in literate people compared to illiterate people. This suggests that written word processing competes with facial processing in the visual cortex of literate individuals (Dehaene et al., 2010). However, these fMRI results cannot address whether the processing facility effects of written language happen simultaneously with the face-related effects during visual processing.

Nevertheless, taking advantage of high temporal resolution, prior event-related potential (ERP) studies can address the above issue. N170 is a negative-going ERP component with a waveform peaking at around 170 ms after stimulus onset (Bötzel et al., 1995; Itier and Taylor, 2004; Caharel et al., 2005; Luo et al., 2010; Li et al., 2013). Additionally, several studies have found that relevant N170 effects can be induced by written language with different processing facilities (Maurer et al., 2006; Maurer et al., 2008; Zhang et al., 2011; Fan et al., 2015) and faces (Bentin et al., 1996; Eimer, 2000a; Liu et al., 2000; Rossion et al., 2000; Fan et al., 2015). This reflects the similar temporal properties of these two kinds of effects. Besides, a human scalp electrophysiological study further investigating the relationship between Chinese character and facial processing found that the Chinese character left-lateralized N170 effect was positively correlated with vocabulary and that the face right-lateralized N170 effect was negatively affected by the character left lateralization and vocabulary. These results demonstrate that the neural development of visual Chinese character processing might compete with that of facial processing (Li et al., 2013).

In order to expand on the findings relevant to object (e.g., Greeble or car) versus face competition (Rossion et al., 2004, 2007), we adopted a competition paradigm to investigate the relationship between Chinese characters from different identification levels and faces during early visual processing in our prior study (Fan et al., 2015). Consistent with the direct competition paradigm of several studies (Rossion et al., 2004, 2007), at the beginning of a trial, a central stimulus was presented. Then, the simultaneous appearance of a central stimulus and a lateralized face led to the competition between their visual processes in our previous study (Fan et al., 2015), which was distinct from the paradigm used in the study (Li et al., 2013) in which Chinese characters and faces were presented in different trials. Therefore, Li et al. (2013) observed the N170 waveforms induced by Chinese characters and faces (both Chinese characters and faces were presented individually), whereas we recorded the N170 waveforms elicited by Chinese characters that were individually presented, faces (contralateral processing) and faces (ipsilateral processing; all faces were simultaneously presented with Chinese characters) in our prior study (Fan et al., 2015). Eventually, we obtained a novel finding of the competition between faces and identifiable Chinese characters during early visual processing, which is reflected by a reduced N170 response to faces that were concurrently presented with identifiable Chinese characters compared with those presented with unidentifiable ones.

However, the time course of the relationship between identifiable Chinese character and house (a non-face stimulus) processing remains unknown. Besides the observation of a significant reduction in VWFA activation for faces, the previous study (Dehaene et al., 2010) found that there was only a marginal decreasing trend toward VWFA activation for houses in literate participants compared to illiterate participants. These results suggest a mainly competitive relationship between written word and facial processing in the VWFA, but not between written word and house processing. Furthermore, the authors of several ERP studies reported that faces evoked a larger N170 than houses (Eimer, 2000b; Rossion et al., 2000) and that inverted faces elicited a larger N170 than upright faces in the unattended condition, but a similar inversion effect was not observed for houses (Feng et al., 2012). These results demonstrate that the neural mechanisms of facial and house processing are categorically distinct, which may lead to different relationships between the processing of Chinese characters and faces and between Chinese characters and houses.

As a follow-up study, we further probed the different relationships between faces and identifiable Chinese characters and between houses (non-face stimuli) and identifiable Chinese characters for a deeper understanding of the processing mechanism of recognizable Chinese characters. We also used the ERP technology and the competition paradigm that is consistent with our previous study (Fan et al., 2015), examining electrophysiological evidence of whether houses compete with identifiable Chinese characters at an early visual processing stage. We hypothesized that the N170 amplitudes elicited by houses would not be significantly reduced when literates concurrently view recognizable Chinese characters and houses compared to trials in which unrecognizable Chinese characters and houses are viewed concurrently. Additionally, we posited our results would replicate our prior findings that after character learning, recognizable characters would induce larger N170 amplitudes than unrecognizable ones; and N170 amplitudes elicited by faces would decline when identifiable Chinese characters and faces are viewed simultaneously compared to trials in which unidentifiable characters and faces are viewed concurrently (Fan et al., 2015). Furthermore, we hypothesized that our results would replicate the previous findings of larger N170 responses for faces than for houses (Eimer, 2000b; Rossion et al., 2000).

## Materials and Methods

### Participants

Sixteen healthy college students (eight males, eight females; age = 19–25 years; mean age = 22.5 years) from Chongqing, China participated in this study as paid volunteers. All participants were native Chinese speakers and had an experience of reading modern Chinese characters (*Song* font characters) for over ten years. None of them have seen *Xiaozhuan* font characters before. They were all right-handed and had normal or corrected-to-normal vision. The study was approved by Human Research Institutional Review Board at Chongqing, University of Arts and Sciences in accordance with the Declaration of Helsinki (1991). The experimental protocol was approved by the Ethics Committee at Chongqing University of Arts and Sciences and informed consents were signed by all participants.

### Materials

#### *Xiaozhuan* Font Characters^1^ (**Figure 1**)

**FIGURE 1:**
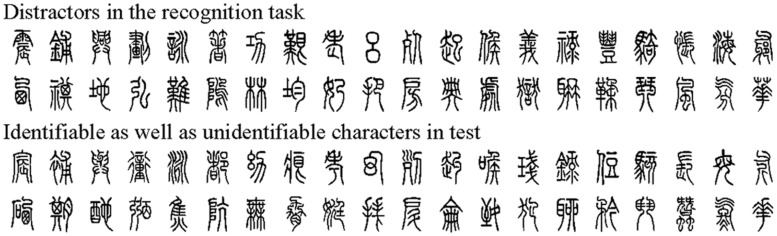
***Xiaozhuan* font characters used in the current study**.

Among the 80 *Xiaozhuan* font characters used in the present study, 20 became identifiable after training, 20 were unidentifiable without training, and 40 were used as distractors in the character recognition task. The 40 characters used for training had been estimated to be unidentifiable by 20 participants who do not participant in our experiment. They were completely distinct in terms of stroke type, which helps prevent a migration effect (i.e., learning any one of the 40 characters cannot influence the learning of the remaining 39 characters as they all were absolutely distinct in the stroke type). In the present study, identifiable characters were unidentifiable characters as well. Hence, the semanteme was controlled and semantic processing might not contribute to the differences between identifiable and unidentifiable conditions. The viewing angle of each image was 3.77 × ×4.27° (when viewed 114 cm from a 17-inch screen whose resolution was 72 pixels per inch). And each black character, whose background color was gray, was presented in the upright, inverted, or mirror form. The character images were similar to one another in luminance (average grayscale value, 6.09 × 10^-5^). Luminance of an image was obtained by averaging the grayscale values of all pixels of an image, which can be measured by MATLAB 2012 software.

#### Combinations of Faces and Characters, and Houses and Characters

Four upright face images were chosen from the native Chinese Facial Affective Picture System (CFAPS; Gong et al., 2011), including two females and two males with neutral expressions. Adobe Photoshop 8.0 software was used to crop each image into the shape of an ellipse, and faces in the images had no makeup, hair, glasses, or beards. In order to control the similarities of physical properties between face and house images, four upright house pictures were cropped into the shape of an ellipse by using Adobe Photoshop 8.0 software. All face and house images were edited with MATLAB 2012 software to be as big as the character pictures. Face and house pictures were similar in luminance (average grayscale value, 6.09 × 10^-5^), and their luminance were similar to that of the characters. By using MATLAB 2012 software, characters were presented in the center of the computer screen, and faces and houses were presented to the left or right of the characters with a horizontal distance of 1.5 cm. Thus, faces were presented 9 cm from the center of the screen (4.52°) as well as houses. All combined pictures were similar in luminance.

### Procedure

During the training, eight participants (four males, four females) learned 20 characters, and the remaining eight participants (four males, four females) learned the remaining 20 characters. There were three stages when participants were trained to learn characters. In stages 1 and 2, participants learned 20 *Xiaozhuan* font characters. In each stage, each participant was trained twice and learned 10 characters. The duration of every training session was 1, 0.25 h, respectively, and there were a 0.5-h break between training sessions, and a 2-h break between stages. In stage 3, it took participants 0.5 h to review the 20 characters. Concrete steps including: At the beginning, participants were asked to if they could identify any of the 20 characters. After that, they were requested to comprehend the pronunciations and meanings of *Xiaozhuan* font characters based on the corresponding *Song* typeface characters. Then, they were requested to perform the writing practice until they reached the standards. Concrete standards included two memory tasks and a recognition task. In two memory tasks, participants were required to write the corresponding *Song* typeface characters (*Xiaozhuan* font characters) accurately, quickly, and consistently based on the corresponding *Xiaozhuan* font characters (*Song* typeface characters). In the recognition task of stage 3, 20 identifiable characters and 20 distractors (10 identifiable characters and 10 distractors in stage 1 or 2) were presented in an upright, inverted, or mirror form for 400 ms each, and after a black screen for 1000–1500 ms, they were required to judge whether they had learned the *Xiaozhuan* font characters that appeared in the center of the screen in an interval of 2 s. For the recognition task, whose accuracy should be more than 95% (No matter which presentation form the character is, one participant can make a mistake in the recognition of one character at the most), participant should identify recognizable characters in the upright form as fast as identifiable characters in inverted and mirror forms.

Before training, all the 16 participants reported that they could not identify any of the 20 characters. After training, in the recognition task, all the participants reached a high accuracy rate (95–100%) on every character, and there was not a significant interaction between the factor “identification level” (identifiable vs. unidentifiable) and the factor “presentation form” (upright, inverted, vs. mirror; *F*_2,30_ = 0.21, *p* = 0.81, $\eta_{p}^{2}$ = 0.014). For recognizable characters, the average reaction times were 297 ms for upright characters, 295 ms for inverted characters, and 298 ms for mirror characters. After character learning, participants who could quickly and accurately read, write, and distinguish the 20 trained *Xiaozhuan* font characters were considered to master the characters.

The experimental procedure was programmed with E-Prime 1.2 (Psychology Software Tools, Inc., Pittsburgh, PA, USA). Our experimental procedure slightly modified the paradigm—at the beginning of each test trial, non-face objects were presented on the center of the screen for 600 ms, and then faces appeared to the left or right of objects for 400 ms with a lateralized face detection task to perform later—used by Rossion et al. (2004) in their study, which has suggested that objects compete with faces during early visual processing in object experts.

In the current experiment, participants were required to fixate on the center of the screen throughout the experiment. At the beginning of every trial, a fixation point was presented with a random duration from 200 to 700 ms. Then, a recognizable or unrecognizable *Xiaozhuan* font characters was presented on the center of the screen for 800 ms, and participants were requested to view it passively during this time. After that, with the character remaining on the screen, a face or a house appeared to the left or right of the character for 400 ms. This duration prevented the N170 waveforms for faces from being affected by visual offset potentials. Although this duration may lead to lateral eye movement, there were few lateral eye movements throughout the experiment, and trials involving them were removed from data analysis.

**FIGURE 2:**
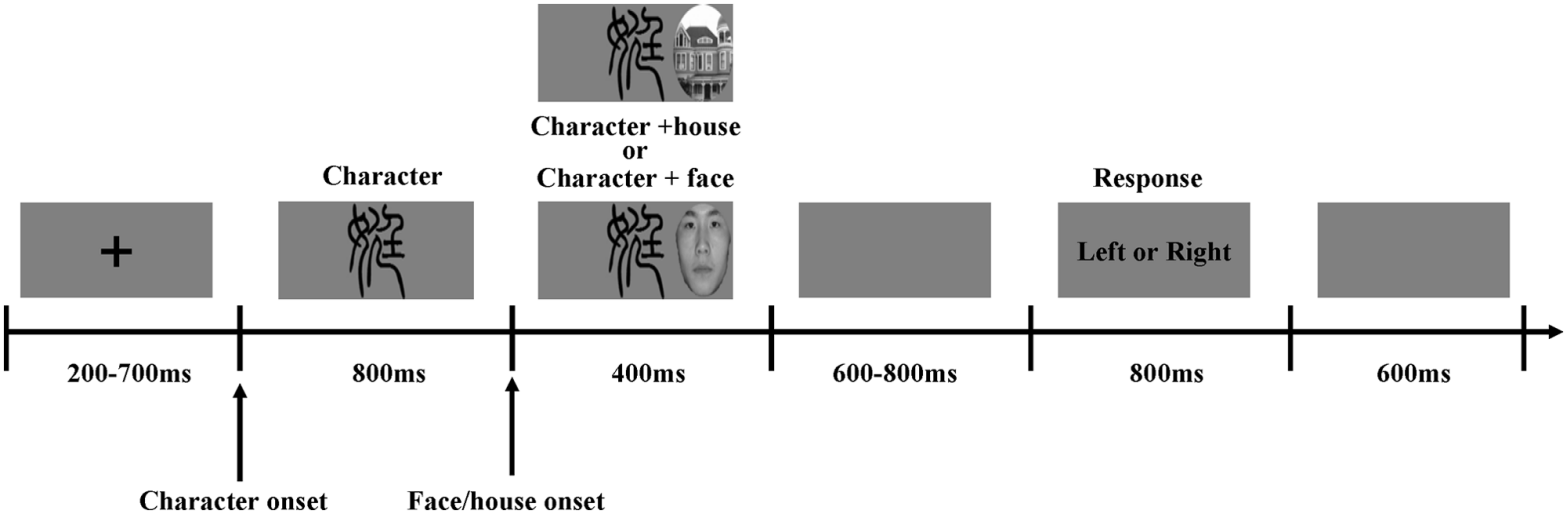
**Overview of a representative experimental trial**. In the beginning, a fixation point was presented for 200–700 ms. Then, a recognizable or unrecognizable *Xiaozhuan* typeface character was presented on the center of the screen for 1200 ms. After an-800 ms-character presentation, a face or a house appeared to the left or right of the character for 400 ms. After the two stimuli disappeared concurrently, there was a 600–800 ms gray screen. Participants were then requested to press keys for responding to the lateralized face/house detection task: pressing “1” when faces or houses appeared on the left side of the characters and pressing “2” when faces or houses appeared on the right side of the characters. Finally, a gray screen was presented for 600 ms.

After the two stimuli disappeared simultaneously, a gray screen was presented for a random duration between 600 and 800 ms. Participants were then requested to press keys for responding to the lateralized face/house detection task, which did not induce any attentional prioritization of any object category (house vs. face), as quickly and accurately as possible, pressing “1” when faces or houses appeared on the left side of the characters and pressing “2” when faces or houses appeared on the right side of the characters. Finally, a gray screen was presented for 600 ms (**Figure 2**). Once the experiment was finished, participants were requested to judge if they could identify the 20 untrained characters, and none of them reported that they could recognize those characters.

The procedure consisted of 480 trials and eight blocks. Each block included 60 trials, and there was a 60–90 s break between blocks. Twenty recognizable and 20 unrecognizable *Xiaozhuan* font characters, respectively, appeared in half of 480 trials. Faces and houses were equivalently presented to the left or right of the characters, leading to 60 trials per condition. Characters appeared randomly so that the participants could not predict the next character, and faces or houses appeared to the left or right of characters in a random order to prevent an expectation effect. Key response was counterbalanced across participants.

### Electroencephalogram Recording and Data Analysis

Based on the extended International 10–20 System, ERPs were recorded from 64 scalp sites in an elastic cap with tin electrodes (Brain Products), with the reference electrode located in the left mastoids. The horizontal electrooculographies (EOGs) were collected from two electrodes situated on the right and left external canthus. The vertical EOGs were collected from two electrodes placed on the infra-orbital and supra-orbital areas of the right eye. All electrode impedances were kept below 5 kΩ. EEG and EOG recordings, which were recorded via a bandpass of 0.01–100 Hz, were sampled at a rate of 500 Hz/channel, and re-referenced oﬄine to acquire a global average.

EEG data was processed oﬄine through Analyzer 2.0 software (Brain Products). The data was corrected for eye-movements and blinks. After that, the data was bandpass filtered at 0.1–30 Hz and segmented with epochs, the duration of which ranged from –200 to 800 ms. After baseline correction (–200 to 0 ms), trials with artifacts were rejected. Trials with eye movements, eye blinks or artifacts exceeding ±50 μV at any electrode were eliminated from ERP averages. Thus, approximately 8% of the trials involving Chinese characters and about 10% of the trials regarding faces and houses were excluded from the average, with the valid trials used for averaging being 440, 218, and 215 for Chinese characters, faces and houses, respectively. Then, EEG data with correct responses was averaged for each condition separately, and there were at least 40 trials to be averaged in each condition. In the previous study (Zhang et al., 2011), P100 peaked maximally at PO7 and PO8, which were chosen to analyze the P100 component elicited by Chinese characters. Besides, several studies (Itier and Taylor, 2004; Brem et al., 2006; Zhang et al., 2011) chose O1 and O2, which were suggested as the source of P100, to analyze the P100 component induced by faces or Chinese characters. Additionally, in some studies (Bentin et al., 1999; Maurer et al., 2005; Brem et al., 2006; Zhang et al., 2011), N170 peaked maximally at the occipito-temporal electrode sites in the left and right hemispheres (PO7 and PO8) which were suggested as the source of N170. Thus, PO7 and PO8 were used for the analysis of the N170 component induced by words in these studies. Apart from PO7 and PO8, previous studies (Luo et al., 2010; Fan et al., 2015; Yi et al., 2015) used P7 and P8 located in the temporal region to analyze the N170 component elicited by faces or Chinese characters. Therefore, according to topographical distribution of grand-averaged ERP activity and previous studies (Bentin et al., 1999; Maurer et al., 2005; Brem et al., 2006; Luo et al., 2010; Zhang et al., 2011; Fan et al., 2015; Yi et al., 2015), we chose PO7/PO8 and O1/O2 to analyze the P100 component, and P7/P8 and PO7/PO8 to analyze the N170 component.

Furthermore, we can understand the nature of an experimental effect by visual inspection of the corresponding waveforms (Picton et al., 2000). Additionally, in some studies (Rossion et al., 2004, 2007; Fan et al., 2015), mean amplitudes induced by central stimuli (Greebles, cars, or Chinese characters), faces (contralateral processing), and faces (ipsilateral processing) were obtained from 30-ms or 40-ms temporal windows that were centered around mean latencies. Thus, we obtained mean amplitudes of P100s/N170s by visual inspection of the grand-averaged figures, which depends on the criteria in the measurement of ERP waveforms and previous studies (Picton et al., 2000; Rossion et al., 2004, 2007; Fan et al., 2015). Mean amplitudes of P100s/N170s were, respectively, induced by characters, faces (contralateral processing), faces (ipsilateral processing), houses (contralateral processing) and houses (ipsilateral processing), (all faces and houses were simultaneously presented with characters). Mean amplitudes of P100s were obtained from five 30-ms temporal windows, respectively, and successively, which were around mean latencies: 85–115 ms, 100–130 ms, 135–165 ms, 95–125 ms, and 130–160 ms. Mean amplitudes of N170s were obtained from five 30-ms temporal windows, respectively, and successively, which were around mean latencies: 155–185 ms, 160–190 ms, 190–220 ms, 150–180 ms, and 185–215 ms.

A three-way repeated measures analysis of variance (ANOVA), examining the N170 component evoked by *Xiaozhuan* font characters, was conducted with the factor “identification level” (two levels: identifiable or unidentifiable), the factor “hemisphere” (two levels: left, right), and the factor “electrode site” (two sites: P7/P8, PO7/PO8). The factors “stimulus category” (face vs. house) and “visual field stimulated” (left, right) were added to the analyses of N170 evoked by faces and houses. The *p*-values were corrected by the Greenhouse–Geisser correction.

## Results

### Behavioral Performance

A three-way repeated measures analyses of variance (ANOVA), examining the accuracies and response times of the task judging the position of laterally presented faces or houses, were conducted with the factor “identification level” (identifiable vs. unidentifiable), the factor “stimulus category” (face vs. house), and the factor “visual field stimulated” (left vs. right). Participants reached a high accuracy rate (88–91%) and response times were between 238 and 261 ms. No matter in terms of task accuracy or task response time, neither significant main effects for all the variables, nor any significant interactions were observed (*p* > 0.1 for all, **Table 1**).

**Table 1:** Behavioral results [Mean (SD)].

	Accuracy (%)				Reaction time (ms)			
	Identifiable		Unidentifiable		Identifiable		Unidentifiable	
	Face	House	Face	House	Face	House	Face	House
Left visual field	90.42 (7.08)	87.92 (8.98)	88.75 (11.28)	89.38 (8.90)	253.20 (46.76)	238.36 (53.97)	244.27 (56.38)	248.16 (55.00)
Right visual field	88.44 (9.20)	89.58 (10.32)	90.31 (7.53)	91.15 (6.69)	243.81 (61.26)	251.38 (55.71)	251.40 (60.28)	261.32 (59.76)

### ERP Data Analysis

#### P100

When it comes to P100 induced by characters, the mean P100 amplitudes did not show a significant main effect for the variable Chinese character identification level (*F*_1,15_ = 0.64, *p* = 0.44, $\eta_{p}^{2}$ = 0.04). In addition, as for P100 elicited by faces or houses, there was no significant interaction between the identification level of Chinese characters and stimulus category on mean P100 amplitude (*F*_1,15_ = 3.00, *p* = 0.10, $\eta_{p}^{2}$ = 0.17).

#### N170

**FIGURE 3:**
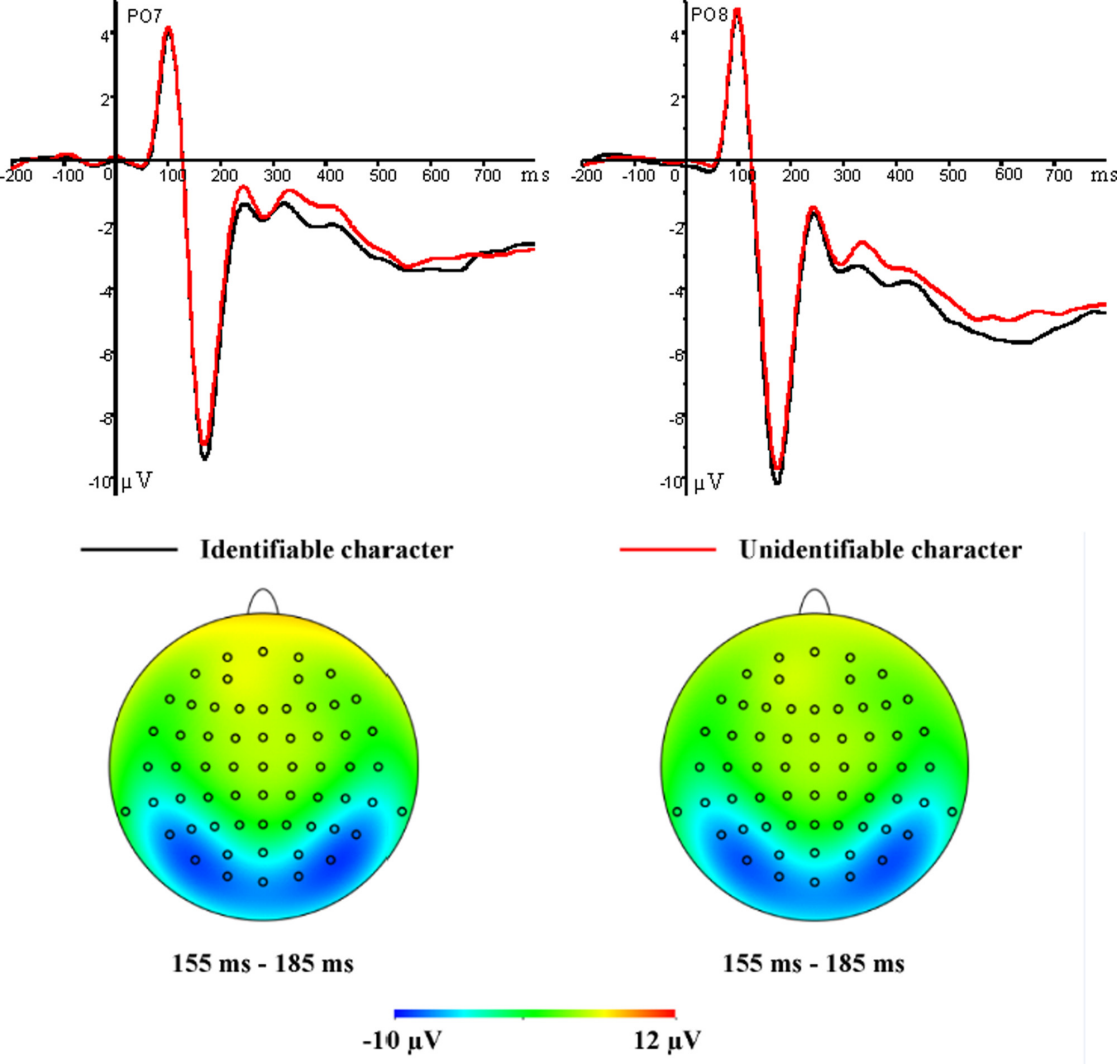
**Grand average ERP waveforms for identifiable character (black lines) and unidentifiable character (red lines) conditions recorded at PO7 and PO8 electrode sites (**Top** row)**. Topographic maps for identifiable characters **(Left)** and unidentifiable characters **(Right)** at 155–185 ms **(Bottom row)**.

In terms of characters, the mean N170 amplitudes showed significant main effects for the variables Chinese character identification level and electrode site (*F*_1,15_ = 5.01, *p* = 0.041, $\eta_{p}^{2}$ = 0.25; *F*_1,15_ = 8.58, *p* = 0.01, $\eta_{p}^{2}$ = 0.36). The pairwise comparison indicated that identifiable characters (–9.40 μV) induced more negative N170 amplitudes than unidentifiable ones (–9.05 μV; **Figure 3**). In addition, larger N170 amplitudes at PO7/PO8 (–9.78 μV) than at P7/P8 (–8.67 μV) were observed.

**FIGURE 4:**
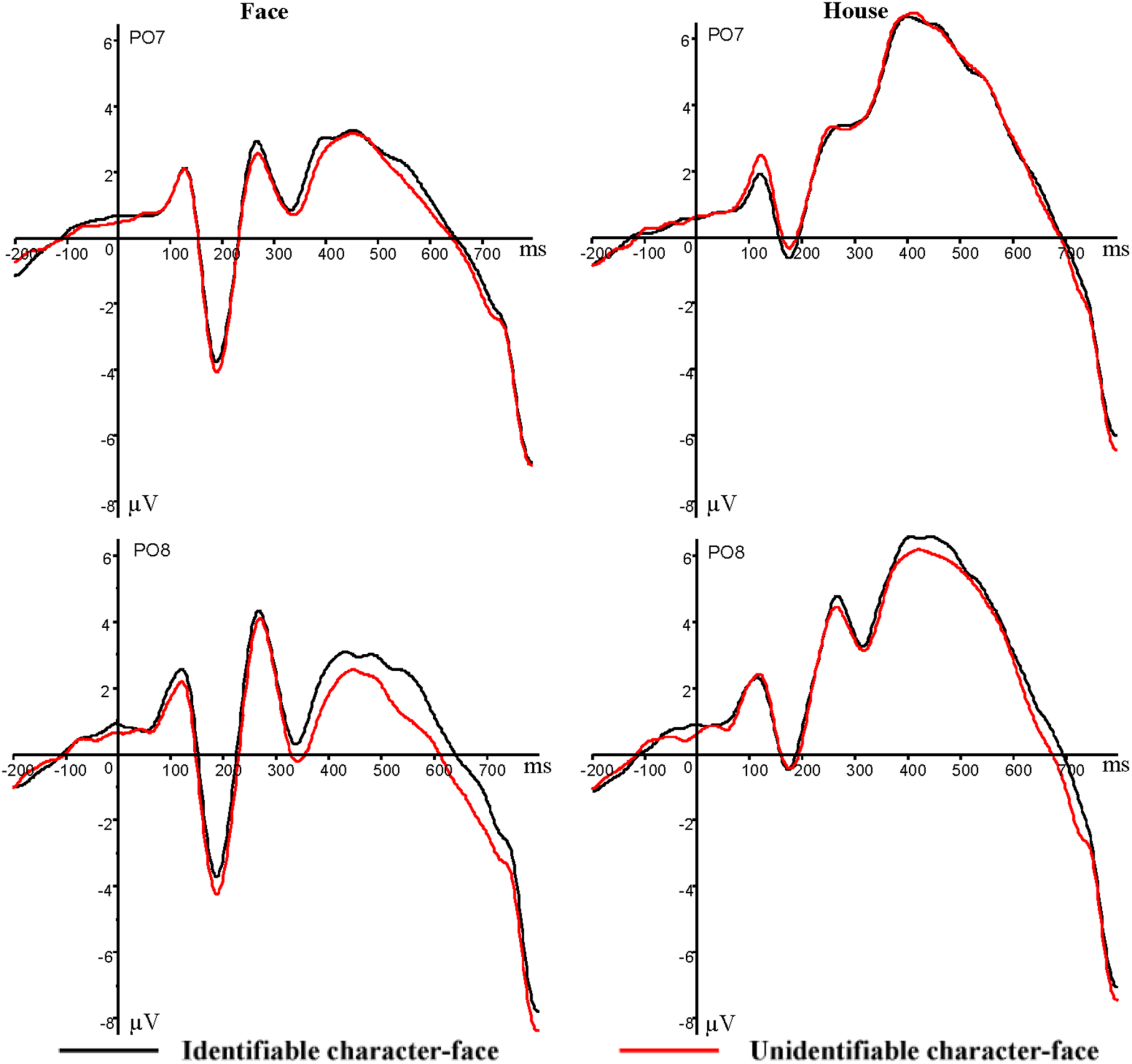
**Grand average ERP waveforms for faces/houses when they were simultaneously viewed with recognizable character (black lines) and when they were simultaneously viewed with unrecognizable character (red lines) recorded at PO7 and PO8 electrode sites**.

**FIGURE 5:**
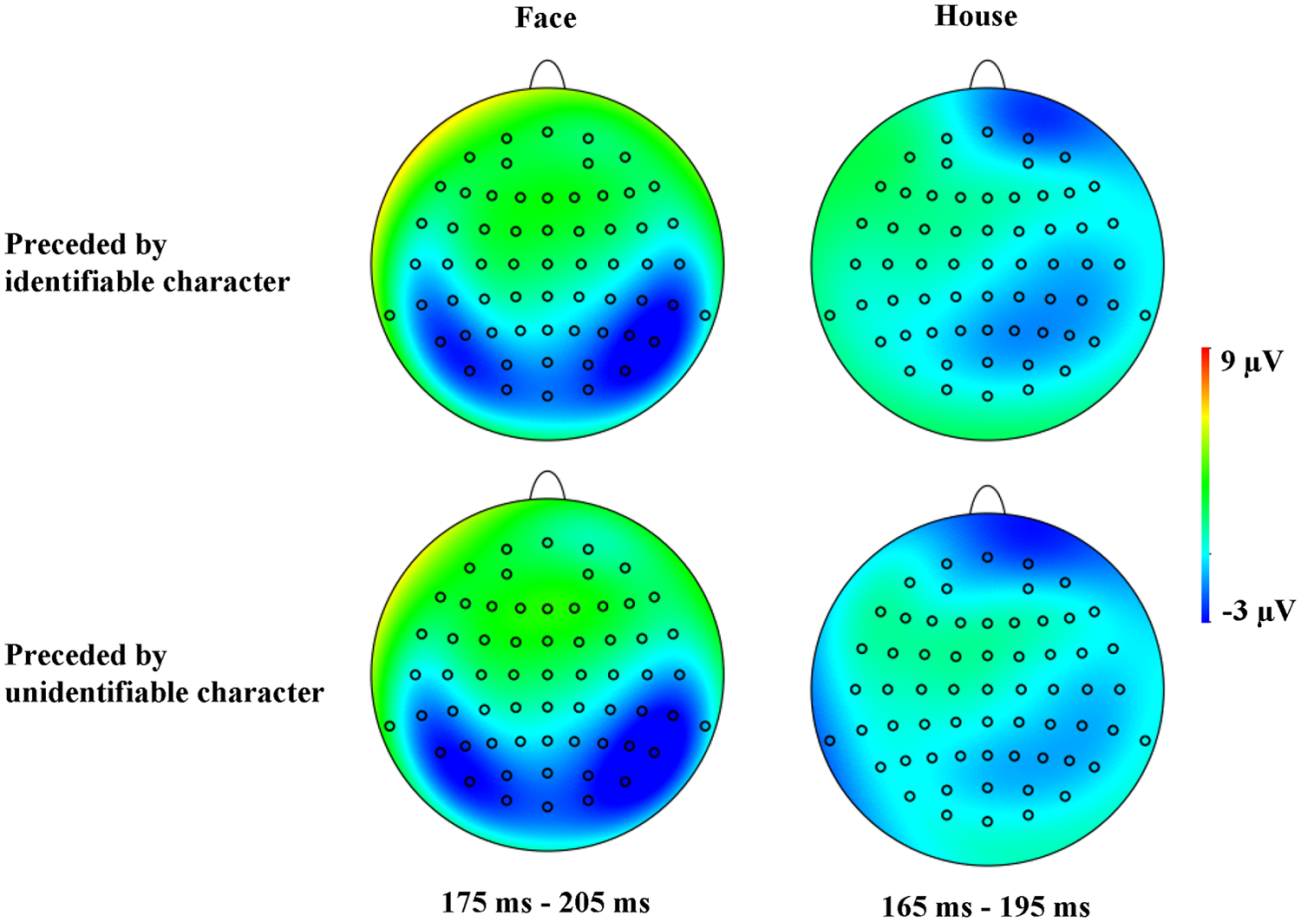
**Topographic maps for faces when they were simultaneously viewed with recognizable or unrecognizable character at 175–205 ms **(Left)** and for houses when they were simultaneously viewed with recognizable or unrecognizable character at 165–195 ms **(Right)****.

In terms of lateralized second stimuli (faces/houses), there was a significant main effect of stimulus category on mean N170 amplitude (*F*_1,15_ = 65.85, *p* = 0, $\eta_{p}^{2}$ = 0.81). The pairwise comparison indicated that faces (–4.96 μV) evoked greater negative N170 amplitudes than houses (–1.05 μV; **Figures 4** and **5**). In addition, there was a significant interaction between the identification level of Chinese characters and stimulus category on mean N170 amplitude (*F*_1,15_ = 4.71, *p* = 0.046, $\eta_{p}^{2}$ = 0.24). Simple effects analyses demonstrated that the N170 amplitudes induced by faces were larger in the unrecognizable character condition (–5.22 μV) compared to the recognizable character condition (–4.71 μV; *p* < 0.05; **Figures 4** and **5**), but the N170 amplitudes evoked by houses did not reach a significant difference between the recognizable character condition (–1.03 μV) and the unrecognizable character condition (–1.06 μV; *p* > 0.05).

Furthermore, the mean N170 amplitudes induced by the second stimuli (faces/houses) showed a significant interaction between the hemisphere and visual field stimulated (*F*_1,15_ = 14.63, *p* = 0.002, $\eta_{p}^{2}$ = 0.49). Simple effects analyses demonstrated that when the second stimuli (faces/houses) were presented in the left visual field, larger N170 amplitudes were observed in the right hemisphere (–4.86 μV) relative to the left hemisphere (–0.63 μV; *p* < 0.05), and when the second stimuli (faces/houses) were presented in the right visual field, greater N170 amplitudes were observed in the left hemisphere (–4.31 μV) compared to the right hemisphere (–2.22 μV; *p* < 0.05), showing that contralateral processing of the second stimuli (faces/houses) elicited larger N170 amplitudes than ipsilateral processing of the same second stimuli (faces/houses).

## Discussion

In the present study, we investigated different relationships between Chinese characters and faces and between Chinese characters and houses at an early visual stage by comparing the associated ERPs in a competition paradigm. We did not observe significant main effects or interactions of the relevant variables on mean P100 amplitude. Therefore, we believe that the N170 effects may not be influenced by overlap with the P100 component. In addition, such competitions are reflected by findings related to ERPs; the N170 amplitudes for faces but not for houses were significantly reduced when they were viewed concurrently with identifiable Chinese characters relative to when they were viewed concurrently with unidentifiable Chinese characters. Therefore, the results indicate that identifiable Chinese characters compete mainly with faces but not houses during early visual processing. Consistent with our previous findings (Fan et al., 2015), we also found that N170 amplitudes were larger for identifiable Chinese characters than for unidentifiable ones, showing that Chinese character identification level modulates N170 amplitude. Additionally, we found that faces induced larger N170 amplitudes than houses, indicating that the neural mechanisms of facial and house processing are different at an early stage (Eimer, 2000b; Rossion et al., 2000; Holmes et al., 2003; Eimer et al., 2010, 2011).

### Faces Compete with Identifiable Chinese Characters During Early Visual Processing

Consistent with our prior study (Fan et al., 2015), we observed that N170 amplitudes induced by faces were larger when they were preceded by unrecognizable Chinese characters compared to recognizable ones. This is due to the same competition paradigm and materials, including faces and *Xiaozhuan* font characters, used in these two studies. Our explanation, which is based on the neuronal recycling hypothesis (Dehaene and Cohen, 2007), is that in our study, at least some parts of the face-related visual processes are progressively recruited for processing Chinese characters after reading training, creating a main competition between identifiable Chinese characters and faces during early visual processing.

### House Processing is Different from Face Processing and Houses Do Not Primarily Compete with Identifiable Chinese Characters During Early Visual Processing

We compared the N170 amplitudes elicited by houses and faces and found that houses induced smaller N170 amplitudes than faces. This finding is consistent with previous results showing the difference between faces and houses during early visual processing (Eimer, 2000b; Rossion et al., 2000; Holmes et al., 2003; Eimer et al., 2010, 2011). More importantly, compared with the condition when houses and unidentifiable Chinese characters were presented concurrently, the N170 amplitudes in response to houses were not significantly reduced when houses and identifiable Chinese characters were presented concurrently.

A prior study (Dehaene et al., 2010) used fMRI to obtain neuroimaging evidence that house processing does not primarily compete with word processing in the VWFA of literate people. Differently, our findings used ERP and the competition paradigm to provide electrophysiological evidence for the hypothesis that house processing may not primarily compete with identifiable Chinese character processing.

Our interpretation is that after short-term character learning, visual processing of identifiable Chinese characters cannot recruit sufficient house-related visual processes; thus, significant competition between the two processes did not occur when houses and identifiable Chinese characters were presented simultaneously. This could be for two reasons. Firstly, people are more visually familiar with faces than less frequently presented visual objects (Schendan et al., 1998; Rossion et al., 2000). To be specific, as faces appear more frequently than houses in our daily life, lower visual familiarity for houses than for faces results in the possibility that insufficient house-related and sufficient face-related visual processes are recruited for visual processing of identifiable Chinese characters. Therefore, identifiable Chinese characters compete primarily with faces rather than houses at an early stage of visual processing. Secondly, whether the Chinese characters are in *Song* or *Xiaozhuan* font, there are some similarities between Chinese characters and faces that do not exist between houses and Chinese characters. Specifically, both faces and Chinese characters are unique in identity, and both are processed based on configural and featural information (Ge et al., 2006; Fu et al., 2012; Li et al., 2013). Li et al. (2013) suggested that these similarities may contribute to a competition between visual Chinese character processing and face-related visual processing. In the present study, our explanation is that these similarities between faces and Chinese characters do not exist between houses and Chinese characters because houses are processed at a category level to a greater extent. Thus, there is no obvious competition between identifiable Chinese characters and houses during early visual processing. Additionally, this may imply that during education, written Chinese characters invade and recycle sufficiently pre-existing face-related processes but insufficient pre-existing house-related processes to supplement the neuronal recycling hypothesis (Dehaene and Cohen, 2007).

There is a limitation in the present study. The presentation time of Chinese characters (800 ms) was limited so that the waveforms induced by Chinese characters did not return to baseline when faces or house were presented. This influenced the waveforms elicited by both faces and houses. However, the waveforms evoked by faces/houses in the baseline did not reach a significant difference between the identifiable character condition and the unidentifiable character condition. Therefore, the identifiable and unidentifiable characters evenly affected the waveforms induced by faces/houses. In our future study, the presentation time of Chinese characters should be longer. Additionally, future work should involve the following points. Firstly, the causes of aforementioned competition are unclear. Therefore, it may be worthwhile to examine which properties of faces, houses, and Chinese characters result in competition (or a lack thereof) between these different categories. Secondly, whether similar results would be observed with higher task load levels is still unknown. A prior study (Mohamed et al., 2009) has suggested that the task load level could modulate the N170 amplitudes elicited by faces. Thus, we aim to examine whether the competitive relationship between the early visual processing of Chinese characters and faces would be modulated by face-related task load level. Finally, it would be interesting to investigate the expertise effect of characters by comparing the N170 components elicited by long-term “naturally developed” character experts versus novices.

## Conclusion

In the present ERP study, we investigated the electrophysiological correlates of the different relationships between the processing of Chinese characters and faces and between Chinese characters and houses at an early visual processing stage. We observed a primary competition between faces and identifiable Chinese characters but not between houses and identifiable Chinese characters during early visual processing, with a significantly reduced N170 for faces but not for houses when they were viewed concurrently with identifiable characters relative to when they were viewed concurrently with unidentifiable characters. Additionally, our results replicate our prior finding of a modulatory effect of Chinese character identification level on the N170 amplitude; there was a significant increase in N170 after characters have been learned. Furthermore, we found an enlarged N170 in response to faces compared to houses, demonstrating that the neural mechanisms for processing faces and houses are different at an early visual processing stage. In summary, these findings further elucidate the neural mechanisms of processing written Chinese characters and provide electrophysiological evidences for the co-processing of different types of visual stimuli in daily life.

## Author Contributions

CF designed the experiment, did this study, analyzed relevant data, and wrote this paper with the help of WL and WH. Additionally, HH, GR, YL, and HL assisted CF in doing the experiment.

## Conflict of Interest Statement

The authors declare that the research was conducted in the absence of any commercial or financial relationships that could be construed as a potential conflict of interest.

## Footnotes

1 *Xiaozhuan* font characters are ancient Chinese characters, which were evolved from Qin state of the Spring and Autumn Period and The Warring States and had been widely used by the end of Western Han. The thickness of strokes of a *Xiaozhuan* font character is consistent, whereas the thickness of strokes of a *Song* font character is different (For instance, the horizontal stroke is thin while the vertical stroke is thick). Additionally, the end of a stroke is pointed in *Xiaozhuan* font, whereas it is round in *Song* font. *Xiaozhuan* font characters used in the current study are different from modern Chinese characters in appearance. Therefore, only people who are engaged in calligraphy and seal cutting can identify, read and write these characters.

## References

[B1] Bentin S. Allison T. Puce A. Perez E. McCarthy G. (1996). Electrophysiological studies of face perception in humans. *J. Cogn. Neurosci.* 8 551–565. 10.1162/jocn.1996.8.6.551 20740065 PMC2927138

[B2] Bentin S. Mouchetant-Rostaing Y. Giard M. H. Echallier J. F. Pernier J. (1999). ERP manifestations of processing printed words at different psycholinguistic levels: time course and scalp distribution. *J. Cogn. Neurosci.* 11 235–260. 10.1162/089892999563373 10402254

[B3] Bolger D. J. Perfetti C. A. Schneider W. (2005). Cross-cultural effect on the brain revisited: Universal structures plus writing system variation. *Hum. Brain Mapp.* 25 92–104. 10.1002/hbm.20124 15846818 PMC6871743

[B4] Bötzel K. Schulze S. Stodieck S. R. (1995). Scalp topography and analysis of intracranial sources of face-evoked potentials. *Exp. Brain Res.* 104 135–143. 7621932 10.1007/BF00229863

[B5] Brem S. Bucher K. Halder P. Summers P. Dietrich T. Martin E. (2006). Evidence for developmental changes in the visual word processing network beyond adolescence. *Neuroimage* 29 822–837. 10.1016/j.neuroimage.2005.09.023 16257546

[B6] Caharel S. Courtay N. Bernard C. Lalonde R. Rebaï M. (2005). Familiarity and emotional expression influence an early stage of face processing: an electrophysiological study. *Brain Cogn.* 59 96–100. 10.1016/j.bandc.2005.05.005 16019117

[B7] Dehaene S. (2005). “Evolution of human cortical circuits for reading and arithmetic: The neuronal recycling hypothesis,” in *Monkey Brain to Human Brain*, eds Dehaene S. Duhamel J. R. Hauser M. Rizzolatti G. (Cambridge, Massachusetts: MIT Press), 133–157.

[B8] Dehaene S. (2009). *Reading in the Brain.* New York, NY: Penguin.

[B9] Dehaene S. Cohen L. (2007). Cultural recycling of cortical maps. *Neuron* 56 384–398. 10.1016/j.neuron.2007.10.004 17964253

[B10] Dehaene S. Pegado F. Braga L. W. Ventura P. Nunes Filho G. Jobert A. (2010). How learning to read changes the cortical networks for vision and language. *Science* 330 1359–1364. 10.1126/science.1194140 21071632

[B11] Dien J. (2009). The neurocognitive basis of reading single words as seen through early latency ERPs: a model of converging pathways. *Biol. Psychol.* 80 10–22. 10.1016/j.biopsycho.2008.04.013 18538915

[B12] Eimer M. (2000a). Effects of face inversion on the structural encoding and recognition of faces: Evidence from event-related brain potentials. *Cogn. Brain Res.* 10 145–158. 10.1016/S0926-6410(00)00038-0 10978702

[B13] Eimer M. (2000b). The face-specific N170 component reflects late stages in the structural encoding of faces. *Neuroreport* 11 2319–2324. 10.1097/00001756-200007140-00050 10923693

[B14] Eimer M. Gosling A. Nicholas S. Kiss M. (2011). The N170 component and its links to configural face processing: A rapid neural adaptation study. *Brain Res.* 1376 76–87. 10.1016/j.brainres.2010.12.046 21172312

[B15] Eimer M. Kiss M. Nicholas S. (2010). Response profile of the face-sensitive N170 component: a rapid adaptation study. *Cereb. Cortex* 20 2442–2452. 10.1093/cercor/bhp312 20080930

[B16] Fan C. Chen S. Zhang L. Qi Z. Jin Y. Wang Q. (2015). N170 changes reflect competition between faces and identifiable characters during early visual processing. *Neuroimage* 110 32–38. 10.1016/j.neuroimage.2015.01.047 25639206

[B17] Feng W. Martinez A. Pitts M. Luo Y.-J. Hillyard S. A. (2012). Spatial attention modulates early face processing. *Neuropsychologia* 50 3461–3468. 10.1016/j.neuropsychologia.2012.09.031 23017595

[B18] Fu S. Feng C. Guo S. Luo Y. Parasuraman R. (2012). Neural adaptation provides evidence for categorical differences in processing of faces and Chinese characters: An ERP study of the N170. *PLoS ONE* 7:e41103 10.1371/journal.pone.0041103 PMC3404057 22911750

[B19] Ge L. Wang Z. McCleery J. P. Lee K. (2006). Activation of face expertise and the inversion effect. *Psychol. Sci.* 17 12–16. 10.1111/j.1467-9280.2005.01658.x 16371138 PMC2575812

[B20] Gong X. Huang Y. X. Wang Y. Luo Y. J. (2011). Revision of the Chinese facial affective picture system. *Chin. Ment. Health J.* 25 40–46.

[B21] Holmes A. Vuilleumier P. Eimer M. (2003). The processing of emotional facial expression is gated by spatial attention: evidence from event-related brain potentials. *Cogn. Brain Res.* 16 174–184. 10.1016/S0926-6410(02)00268-9 12668225

[B22] Itier R. J. Taylor M. J. (2004). N170 or N1? Spatiotemporal differences between object and face processing using ERPs. *Cereb. Cortex* 14 132–142. 10.1093/cercor/bhg111 14704210

[B23] Li S. Lee K. Zhao J. Yang Z. He S. Weng X. (2013). Neural competition as a developmental process: early hemispheric specialization for word processing delays specialization for face processing. *Neuropsychologia* 51 950–959. 10.1016/j.neuropsychologia.2013.02.006 23462239 PMC3756286

[B24] Liu J. Higuchi M. Marantz A. Kanwisher N. (2000). The selectivity of the occipitotemporal M170 for faces. *Neuroreport* 11 337–341. 10.1097/00001756-200002070-00023 10674482

[B25] Luo W. Feng W. He W. Wang N. Y. Luo Y. J. (2010). Three stages of facial expression processing: ERP study with rapid serial visual presentation. *Neuroimage* 49 1857–1867. 10.1016/j.neuroimage.2009.09.018 19770052 PMC3794431

[B26] Maurer U. Brandeis D. McCandliss B. D. (2005). Fast, visual specialization for reading in English revealed by the topography of the N170 ERP response. *Behav. Brain Funct.* 1:13 10.1186/1744-9081-1-13 PMC1208852 16091138

[B27] Maurer U. Brem S. Kranz F. Bucher K. Benz R. Halder P. (2006). Coarse neural tuning for print peaks when children learn to read. *Neuroimage* 33 749–758. 10.1016/j.neuroimage.2006.06.025 16920367

[B28] Maurer U. Zevin J. D. McCandliss B. D. (2008). Left-lateralized N170 effects of visual expertise in reading: evidence from Japanese syllabic and logographic scripts. *J. Cogn. Neurosci.* 20 1878–1891. 10.1162/jocn.2008.20125 18370600 PMC4416222

[B29] McCandliss B. D. Cohen L. Dehaene S. (2003). The visual word form area: expertise for reading in the fusiform gyrus. *Trends Cogn. Sci.* 7 293–299. 10.1016/S1364-6613(03)00134-7 12860187

[B30] Mohamed T. N. Neumann M. F. Schweinberger S. R. (2009). Perceptual load manipulation reveals sensitivity of the face-selective N170 to attention. *Neuroreport* 20 782–787. 10.1097/WNR.0b013e32832b7e24 19369907

[B31] Picton T. Bentin S. Berg P. Donchin E. Hillyard S. Johnson R. (2000). Guidelines for using human event-related potentials to study cognition: recording standards and publication criteria. *Psychophysiology* 37 127–152. 10.1111/1469-8986.3720127 10731765

[B32] Rossion B. Collins D. Goffaux V. Curran T. (2007). Long-term expertise with artificial objects increases visual competition with early face categorization processes. *J. Cogn. Neurosci.* 19 543–555. 10.1162/jocn.2007.19.3.543 17335400

[B33] Rossion B. Gauthier I. Tarr M. J. Despland P. Bruyer R. Linotte S. (2000). The N170 occipito-temporal component is delayed and enhanced to inverted faces but not to inverted objects: an electrophysiological account of face-specific processes in the human brain. *Neuroreport* 11 69–72. 10.1097/00001756-200001170-00014 10683832

[B34] Rossion B. Kung C.-C. Tarr M. J. (2004). Visual expertise with nonface objects leads to competition with the early perceptual processing of faces in the human occipitotemporal cortex. *Proc. Natl. Acad. Sci. U.S.A.* 101 14521–14526. 10.1073/pnas.0405613101 15448209 PMC521961

[B35] Schendan H. E. Ganis G. Kutas M. (1998). Neurophysiological evidence for visual perceptual categorization of words and faces within 150 ms. *Psychophysiology* 35 240–251. 10.1111/1469-8986.3530240 9564744

[B36] Schroer R. (2008). Open your eyes and look harder!(An investigation into the idea of a responsible visual search). *South. J. Philos.* 46 409–430. 10.1111/j.2041-6962.2008.tb00126.x

[B37] Slagter H. A. Lutz A. Greischar L. L. Francis A. D. Nieuwenhuis S. Davis J. M. (2007). Mental training affects distribution of limited brain resources. *PLoS Biol.* 5:e138 10.1371/journal.pbio.0050138 PMC1865565 17488185

[B38] Thesen T. McDonald C. R. Carlson C. Doyle W. Cash S. Sherfey J. (2012). Sequential then interactive processing of letters and words in the left fusiform gyrus. *Nat. Commun.* 3:1284 10.1038/ncomms2220 PMC4407686 23250414

[B39] Yi S. He W. Zhan L. Qi Z. Zhu C. Luo W. (2015). Emotional Noun Processing: an ERP study with rapid serial visual presentation. *PLoS ONE* 10:e0118924 10.1371/journal.pone.0118924 PMC4349822 25738633

[B40] Zhang M. Jiang T. Mei L. Yang H. Chen C. Xue G. (2011). It’s a word: Early electrophysiological response to the character likeness of pictographs. *Psychophysiology* 48 950–959. 10.1111/j.1469-8986.2010.01153.x 21091960

